# Co-creation using crowdsourcing to promote PrEP adherence in China: study protocol for a stepped-wedge randomized controlled trial

**DOI:** 10.1186/s12889-022-14117-5

**Published:** 2022-09-07

**Authors:** Yongjie Sha, Chunyan Li, Yuan Xiong, Aniruddha Hazra, Jonathan Lio, Ivy Jiang, Haojie Huang, Jared Kerman, Jannelle Molina, Linghua Li, Ke Liang, Dandan Gong, Quanmin Li, Songjie Wu, Renslow Sherer, Joseph D. Tucker, Weiming Tang

**Affiliations:** 1 University of North Carolina at Chapel Hill Project-China, 7 Lujing Road, Guangzhou, 510091 Guangdong Province China; 2 Department of Health Behavior, Gillings School of Global Public Health, University of North Carolina at Chapel Hill, Chapel Hill, North Carolina USA; 3 Section of Infectious Diseases and Global Health, Department of Medicine, 5841 S. Maryland Avenue, Chicago, IL 60637 USA; 4 Wuhan LGBTQ Center, Wuhan, Hubei China; 5 Gilead Sciences, Foster City, California USA; 6 Department of Infectious Diseases, Guangzhou Number Eight People’s Hospital, Guangzhou, China; 7 Department of Infectious Diseases, Zhongnan Hospital of Wuhan University, Wuhan, China; 8 Institute of Global Health and Infectious Diseases, University of North Carolina at Chapel Hill, Chapel Hill, North Carolina USA; 9 Faculty of Infectious and Tropical Diseases, London School of Hygiene and Tropical Medicine, London, UK

**Keywords:** Co-creation, PrEP, Adherence, China, GBMSM, Transgender, Stepped-wedge design, Randomized controlled trial

## Abstract

**Background:**

Adherent pre-exposure prophylaxis (PrEP) uptake can prevent HIV infections. Despite the high HIV incidence, Chinese key populations have low PrEP uptake and adherence. New interventions are needed to increase PrEP adherence among key populations in China. Co-creation methods are helpful to solicit ideas from the community to solve public health problems. The study protocol aims to describe the design of a stepped-wedge trial and to evaluate the efficacy of co-created interventions to facilitate PrEP adherence among key populations in China.

**Methods:**

The study will develop intervention packages to facilitate PrEP adherence among Chinese key populations using co-creation methods. The study will then evaluate the efficacy of the co-created intervention packages using a stepped-wedge randomized controlled trial. This four-phased closed cohort stepped-wedge design will have four clusters. Each cluster will start intervention at three-month intervals. Seven hundred participants who initiated PrEP will be recruited. Participants will be randomized to the clusters using block randomization. The intervention condition includes receiving co-created interventions in addition to standard of care. The control condition is the standard of care that includes routine clinical assessment every 3 months. All participants will also receive an online follow-up survey every 3 months to record medication adherence and will be encouraged to use a WeChat mini-app for sexual and mental health education throughout the study. The primary outcomes are PrEP adherence and retention in PrEP care throughout the study period. We will examine a hypothesis that a co-created intervention can facilitate PrEP adherence. Generalized linear mixed models will be used for the primary outcome analysis.

**Discussion:**

Developing PrEP adherence interventions in China faces barriers including suboptimal PrEP uptake among key populations, the lack of effective PrEP service delivery models, and insufficient community engagement in PrEP initiatives. Our study design addresses these obstacles by using co-creation to generate social media-based intervention materials and embedding the study design in the local healthcare system. The study outcomes may have implications for policy and intervention practices among CBOs and the medical system to facilitate PrEP adherence among key populations.

**Trial registration:**

The study is registered in Clinical Trial databases in China (ChiCTR2100048981, July 19, 2021) and the US (NCT04754139, February 11, 2021).

**Supplementary Information:**

The online version contains supplementary material available at 10.1186/s12889-022-14117-5.

## Background

Despite many control programs, HIV incidence is still high among key populations in China. The national prevalence of HIV from 2001 to 2018 among gay, bisexual and other men who have sex with men (GBMSM) was estimated to be 5.7% (95% CI: 5.4–6.1%) [1]. Sexual transmission has become the preeminent route for HIV infection since 2014, with 95% of cases reportedly due to sexual transmission [2, 3]. The proportion of HIV cases due to heterosexual contact increased from 50% in 2010 to over 70% in 2018 [4]. HIV prevalence among Chinese MSM rose from 1.5% in 2005 to 7.0% in 2018 [4, 5]. The evolving patterns of HIV infections in China require new interventions.

Pre-exposure prophylaxis (PrEP) is highly effective in preventing new HIV infections among adherent key populations [6–8]. However, suboptimal adherence to PrEP in real-world studies is a substantial barrier [9, 10]. In China, TDF-FTC was only approved in August 2020 for PrEP. PrEP access is limited to urban infectious disease hospitals and not covered by national health programs or major health insurance plans. As a result, PrEP awareness and uptake are still low among Chinese key populations [11, 12], and data on PrEP adherence is scarce. Using self-reported adherence to daily PrEP, a study among 584 MSM in western China suggested a median adherence rate of 64.3% in 2018 [13]. The low engagement in the PrEP care continuum among key populations calls for innovative efforts to promote PrEP use. In particular, as PrEP moves into routine HIV prevention care practice and China is taking steps to scale up PrEP uptake, optimizing medication adherence becomes increasingly critical to fully realize its potential. Prior interventions have used mobile phone applications, text messages, and incentives to increase PrEP adherence among key populations in global settings [14–17]. However, these interventions were not tailored to the focus community or were based on sociocultural settings that were less suitable for key populations in China.

Crowdsourcing is a method of co-creation that uses a bottom-up approach to solicit ideas from groups of people to solve all or part of a practical problem [18, 19]. Crowdsourcing has been increasingly implemented to develop public health interventions [20]. Data from our previous study suggested that video campaigns generated through crowdsourcing can effectively strengthen and expand community-based HIV testing services for Chinese MSM [21]. Compared to conventional approaches, crowdsourcing may facilitate community engagement, while creating novel solutions to public health problems that resonate with the community [22]. In particular, crowdsourcing can help engage vulnerable groups who are often excluded from research projects [23]. However, most crowdsourced interventions in sexual health research centered on HIV testing services and promoting sexual health programs [24]. Preliminary data from pilot programs suggest that crowdsourcing methods could be used to promote PrEP uptake [24].

The study proposal aims to describe the design of a stepped-wedge trial and to evaluate the efficacy of a crowdsourced intervention to facilitate PrEP adherence among at-risk populations in China.

## Trial aims

Our study will use a crowdsourcing open call to develop a PrEP adherence intervention that will be implemented and evaluated using a stepped-wedge cluster randomized trial. The aim of this stepped-wedge trial is to improve PrEP adherence through crowdsourced intervention. We hypothesize that a crowdsourced intervention can facilitate PrEP adherence.

## Methods/design

### Study design and rationale

This pragmatic stepped-wedge cluster randomized trial is a sub-study of a multi-center demonstration of PrEP engagement and initiation among at-risk populations in two Chinese provinces, Hubei and Guangdong. (Study ID: CO-US-276-5866) In the demonstration, participants recruited from both sites will be provided with daily oral PrEP for 12 months with quarterly follow-up visits (questionnaires and clinical assessments). Participants are encouraged to adhere to daily regimen throughout the study. At enrollment, every participant will be given access to a WeChat-based PrEP education mini-app that has been pilot tested and refined in a previous study project among Chinese GBMSM [25]. Among the participants who successfully initiate PrEP through the study, the first 700 participants from both sites will be included in the stepped-wedge trial.

This stepped-wedge design will be a four-phased implementation organized into four clusters with each cluster starting the intervention at three-month intervals, so that by the end of the 12-month study period all clusters will receive the intervention. (Table 1) A pragmatic trial is aligned with the aim of our study as we are interested in examining the crowdsourced intervention in a real-world context rather than strictly measuring its efficacy in an ideal setting [26]. This stepped-wedge design allows the evaluation of the intervention’s external validity and informs public health decisions to promote PrEP adherence in at-risk populations [27].

**Table 1:**
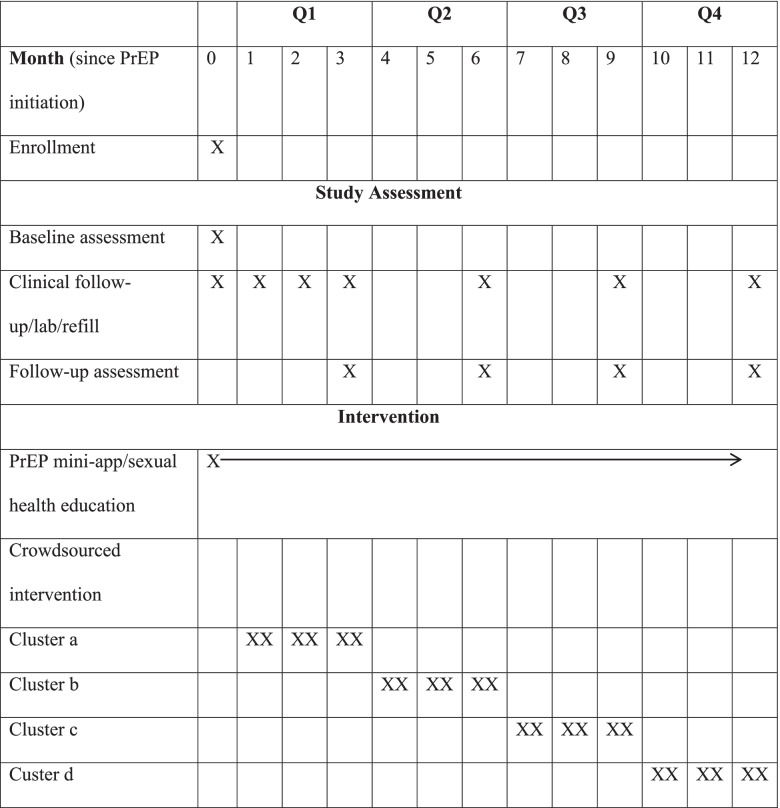
Stepped-wedge design of a crowdsourced intervention for facilitating PrEP adherence in at-risk populations in China

In this closed cohort stepped-wedge design, all participants will be identified at the onset of the trial. All participants will be engaged with the intervention or control condition at different crossover points. Minimal implementation lag will be expected as the predesigned interventions consist of social media components and immediately start and finish [22]. The control condition includes routine follow-up visits (surveys and clinical assessments) by community-based organizations and local hospitals following the setup of the demonstration study. The intervention condition includes a crowdsourced intervention to facilitate PrEP adherence.

### Eligibility

Eligibility criteria for participants in the intervention include: 1) 18 years old or older at the time of enrollment; 2) Chinese citizens, currently residing in Hubei or Guangdong province and planning to remain there for the next 12 months; 3) Having a smartphone with WeChat installed; 4) Must self-report at least one of the listed high-risk behaviors (a. Have sex with more than 1 partners in the past 90 days; b. Inconsistent condom usage in vaginal or anal sex in the past 6 months; c. Ongoing sexual relationships with an HIV-positive partner in the past 6 months; d. Participation in commercial sex work in the past 6 months; e. Any bacterial STI diagnosed or reported in the past 6 months); 5) No history of renal dysfunction; 6) No history of chronic hepatitis B (HBV) infection; 7) No HIV infection; 8) No indication for HIV post-exposure prophylaxis; 9) No signs or symptoms consistent with acute HIV infection.

Potential participants will be informed of this stepped-wedge trial when they initiate PrEP in the hospital by study volunteers. Signed informed consent will be required from all participants.

### Sample size

A total of 700 participants will be recruited from Hubei and Guangdong provinces. The sample size of this closed cohort, binary outcome, complete stepped-wedge RCT design was calculated using PASS Sample Size Software (NCSS LLC, Kaysville, Utah). The required sample size was calculated for the primary outcome. We assumed that a crowdsourced intervention will facilitate PrEP adherence among at-risk populations compared to the standard routine follow-up. This calculation was based on the Hussey and Hughes model postulating the cluster-specific departure from the average is homogeneous across time periods and intervention sequences [28, 29]. We assumed an intra-cluster correlation coefficient (ICC) of 0.02. Assuming an intervention effect of 0.2 (0.6 under intervention, 0.4 under control), a total of four clusters, four total intervention time periods. Two-sided Wald Z-Test alpha = 0.05, 80% power, the total sample size was 540. Accounting for 20% lost to follow-up, we increased the total sample size to 700 (175 for each cluster).

### Intervention

The intervention consists of two parts: 1) intervention materials delivered to participants through WeChat in a stepped-wedge cluster randomized trial; 2) a series of judging by all participants on materials co-created by a small group of participants. The intervention effect will take into consideration of the two aspects. Intervention materials are developed through a nationwide open call in China.

### Intervention materials development – crowdsourcing open call

**Fig. 1:**
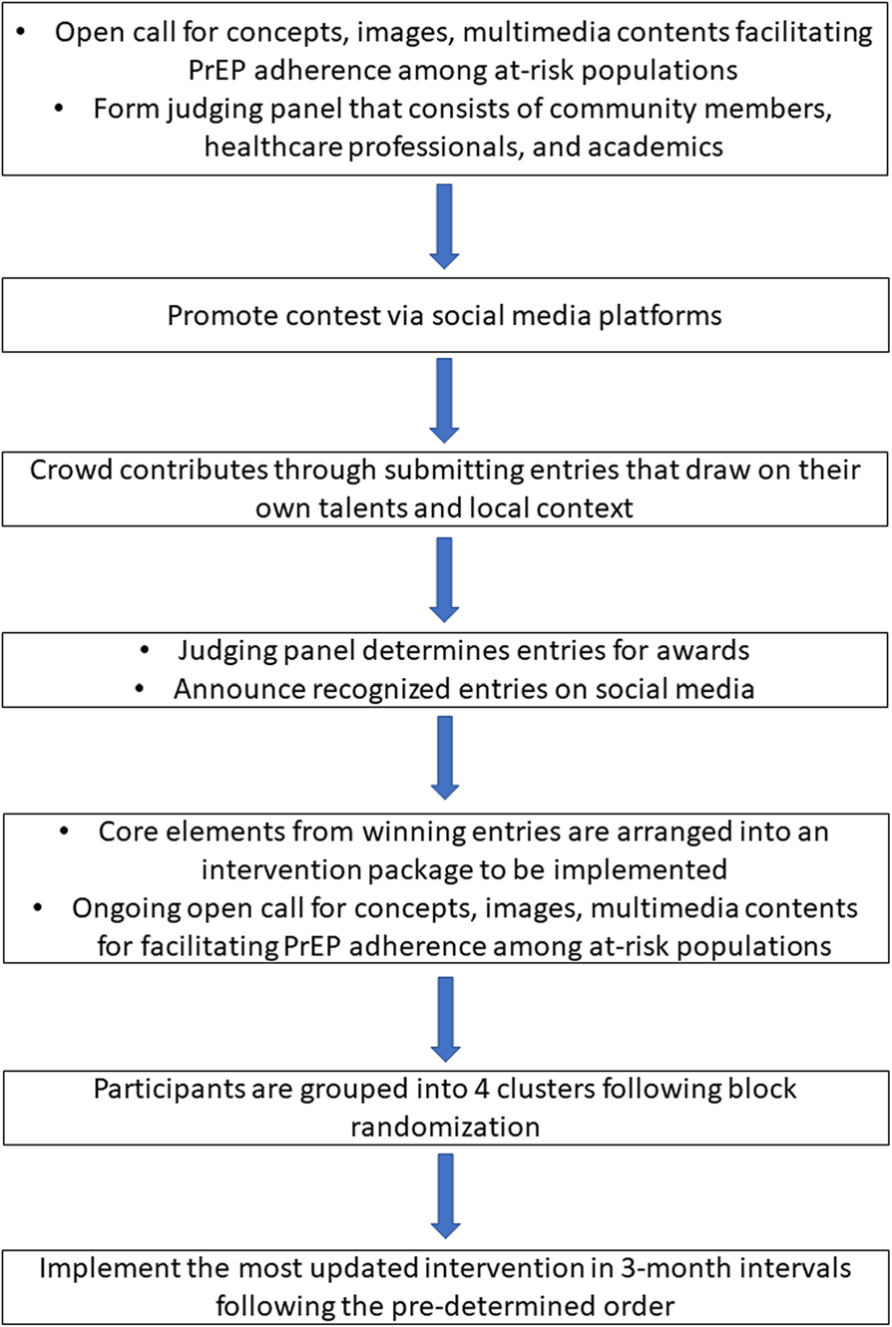
Schematic of crowdsourcing intervention development and implementation

The intervention has been developed from a national crowdsourcing open call (Fig. 1). This crowdsourcing open call focused on identifying submissions to promote PrEP adherence among at-risk populations. Eligible submissions included concepts (< 1000 characters), images (photographs, posters, drawings), and multimedia contents (videos, audios, H5). All announcements related to the open call (promotions, deadlines, prizes) were channeled through social media platforms including WeChat and Weibo.

As of September 2021, the open call has received 19 submissions and each was evaluated by at least three independent judges. The judging panel consisted of CBO members, healthcare professionals and academics. Judges were selected based on their expertise in relevant sexual health topics and crowdsourcing. The quality of the entries was judged on relevance, feasibility, and elaboration. Judges received a full description of the judging criteria in advance and provided a single score on a 10-point scale. Following these judging criteria, all entries were screened to check for relevance to our open call and to identify plagiarism. People who submitted contributions that were determined to be in the top three were awarded cash prizes. Given that participation is important for crowdsourcing, the open call will keep running during the trial. This will allow participants to develop messages and ideas to iteratively enhance the intervention. The research team will also update the intervention by integrating selected materials from the ongoing open call every 3 months following the initial intervention (1.0). Materials will be adapted to formats compatible with the delivering platform WeChat.

Among the 19 submissions, eight were texts or slogans, two were videos, nine were posters, photos or comics. Five posters and one comic were selected by the judging panel as the most recognized contributions (see Supplement). The initial intervention (1.0) will integrate images from the six contributions. The themes of these images are focused on raising PrEP awareness by emphasizing the effectiveness and protective aspects of PrEP or on increasing PrEP knowledge in terms of who should take PrEP and the two types of PrEP medications. Core elements used in these images included posters, infographics, and comics.

The initial intervention (1.0) will integrate the recognized contributions for messages to encourage PrEP adherence. These materials will be combined with adjustments by the study team to allow for clear messaging. The intervention package will be channeled biweekly through WeChat and will allow for multiple deliveries during the intervention period. The intervention implementation will incorporate CBO peer educators or volunteers in Wuhan and Guangzhou as peer educators have access to the social media platform and are in close contact with the participants. CBO peer educators will be responsible for recruiting participants, following up with participants throughout the study period of 12 months, and implementing the intervention.

### Stepped-wedge cluster randomized trial design

The intervention materials developed through the crowdsourcing open call will be delivered in a stepped-wedge cluster randomized trial. This stepped wedge design will be a four-phased implementation (four clusters) with each cluster starting the intervention at three-month intervals (Table 1).

### Cluster randomization

Block randomization will be used to control for potential secular trends and bias related to the imbalance in cluster-level covariates [30]. Block size will be eight. Participants at each site will be assigned to blocks based on the timing of PrEP initiation. Participants enrolled at the same site will be grouped to the same block. Blocks will be assigned to the four clusters using a computer-generated randomization list that is generated by a study staff member.

### Intervention condition - receiving the intervention materials

Participants will be assigned into four clusters in blocks at each site. All clusters will receive the intervention initiated at a different time. Updated interventions 2.0, 3.0, 4.0 will be implemented for clusters b, c, d, respectively, in addition to cluster a receiving intervention 1.0. Each cluster will receive the intervention for 3 months over a 12-month period. The first intervention will start 2 weeks after the baseline recruitment. The time intervals of the crowdsourced intervention are aligned with the follow-up intervals in the PrEP Demo study. That is, the first cluster will receive their first intervention 2 weeks after they fill out the baseline survey, and the second, third, and fourth cluster will receive their first intervention 2 weeks after they complete the follow-up surveys at 3, 6, 9 months respectively. The intervention will be delivered to each cluster biweekly in the 3-month intervention period by the CBOs. CBO peer educators or volunteers will contact participants via WeChat and implement the intervention each time.

### Intervention condition - judging and co-creation group

Along with the crowdsourced interventions, participants in the intervention condition will be additionally engaged by a series of judging on materials co-created by a small group of participants. This small co-creation group will be selected from trial participants based on their willingness to contribute and areas of expertise while also considering their self-identified gender and sexual identity. The co-creation group will generate materials regularly to address adhering to PrEP. Co-creations that are only tailored to the experience of one population will be tweaked by the study group before sending to other populations in the sample to better reflect the latter’s specific experience. The co-created materials will be sent to all participants in the intervention condition for judging. The judging criteria will include 1) potential to encourage PrEP adherence; 2) creativity; 3) potential to engage social media users; 4) feasibility for real-world implementation. Materials that have the highest scores will be shared publicly on social media platforms.

### Control condition

When clusters are not in the intervention condition, they will be in the control condition. Control condition includes a baseline program using WeChat mini-app to promote sexual and mental health awareness and routine follow-ups by CBOs and hospitals. This mini-app promoting sexual and mental health awareness among Chinese gender and sexual minorities is predesigned by the research team and will be directly accessed on WeChat mini-app store for free [25]. The mini-app comprises synthesized LGBTQ-friendly information on PrEP (what is PrEP, benefits of PrEP, who can use PrEP, how to use PrEP, etc.) and other sexual and mental health-related content, and allows participants to apply for free HIV self-tests and communicate with study staff members directly. Participants will be introduced to the mini-app at baseline recruitment and will be encouraged to use the mini-app throughout the study. Participants will also receive follow-ups from the hospitals and CBOs at 3-month intervals to assess PrEP uptake and adherence. The follow-ups include an online follow-up survey to track PrEP adherence and a clinical assessment for ongoing PrEP uptake. The follow-up survey will be administered by CBO peer educators or volunteers, who will be responsible for sending out the survey and collecting survey responses. The clinical assessment will be performed by experienced physicians in the hospitals following standard guidelines.

### Study measures and outcomes

A baseline survey will collect data related to sociodemographic characteristics, sexual behaviors, experiences and perceptions of PrEP, sociopsychological assessments related to PrEP use, and co-creation willingness. Sociodemographic characteristics include participants’ age, gender identity, sexual orientation, socioeconomic status, medical insurance status, and housing. Sexual behaviors variables include number of sexual partners, casual sex in the past 6 months, sex work in the past 6 months, sex under the influence of substance (including stimulants or poppers, alcohol, cocaine or speed/crystal meth) in the past 6 months, HIV testing, STI testing, and self-assessed HIV infection risks. Experiences and perceptions of PrEP include awareness, willingness, preferences, previous use of PrEP, PrEP attitudes, PrEP stigma, and PrEP use self-efficacy. Sociopsychological assessments include HIV-related anxiety, social support, and overall perceived stress. Co-creation willingness asks whether the participant would be willing to join a co-creation group, and their areas of expertise if they agree to. Participation in the PrEP open call will also be captured. For self-identified transgender individuals, data related to gender-affirming interventions, lifetime involvement in sex work, and community connectedness will also be collected. Baseline and follow-up surveys will be built online using Sojump (Sojump, Shanghai, China).

Each follow-up at the 3, 6, 9, 12 months will include an online survey and a clinical assessment. (Table 1) The online survey will collect data related to number of sexual partners, HIV testing, PrEP uptake (missing doses in the past 90, 30, 7 days for daily PrEP users vs. number of sex and number of doses taken in the past 90, 30 days for on-demand PrEP users), engaging in WeChat mini-app, and evaluation of crowdsourced PrEP adherence intervention. Participants will be assessed on their PrEP attitudes, PrEP stigma, PrEP use self-efficacy, HIV-related anxiety, social support and overall perceived stress using validated scales at 6 and 12 months. For all follow-ups, transgender participants will additionally respond to questions related to whether they are currently using gender affirming hormones, and if they were, whether they are concerned about drug-drug interaction and whether they have mentioned taking hormones to the doctors who are prescribing PrEP. The clinical assessment will include testing results related to HIV, STIs (syphilis, HBV, HCV), standard blood test, standard urine test, liver function test, and renal function test. If a participant seroconverts to HIV positive, their participation in the study will be terminated and they will be referred to their local provider or health department for antiretroviral therapy. Otherwise, participants will be prescribed TDF-FTC for another 3 months after each follow-up at 3, 6, 9 months.

The primary outcomes of this sub-study are PrEP adherence and retention in PrEP care throughout the study period. PrEP adherence will be measured by self-report in quarterly behavioral assessment surveys and pill count at clinical follow-ups among a subset (a total of 120 participants who initiate PrEP during the study in Guangdong will be sampled to receive pill count using systematic random sampling). Retention in PrEP care will be measured via self-initiated or physician-advised PrEP discontinuation, PrEP re-initiation, and self-initiated switch rates from once daily and on-demand regimens. Details about the study measures are illustrated in Table 2. Optimal daily PrEP adherence will be considered to be 6–7 pills weekly. Optimal adherence to an on-demand regimen will be defined as having two pills at least 2 h before each sexual encounter, followed by one pill 24 hours after the first dose and one pill after the second dose.

**Table 2:** Outcome measures and timepoints of data collection

Outcomes	Measure	Data collection timepoints
**Primary outcomes**		
**Retention in PrEP care**	Number of participants completed clinical follow-ups	3, 6, 9, 12 months
**PrEP adherence**	1. Self-report in follow-up surveys: During the past 7 days and 30 days: (1) self-report taking once daily regimen: number of missed pills; perceptions of whether they had taken < 60%, 60–90%, or > 90% doses. (2) self-report taking on-demand regimen: number of sex events that are not covered by any PrEP (0 pills taken); correctly covered by PrEP (2 pills on the sex day (X) and X + 1, X + 2; or at least one pill taken on days X, X + 1, and X + 2 if the days between two sex events are less than one week); partially covered by PrEP (any other use). 2. Pill count at clinical visits: number of unused pills counted at each clinical follow-up (in-person or via phone) 3. Medical possession ratio: [(total days between first fill and end of follow-up) – (number of days without TDF-FTC in possession)] / total days of follow-up, based on medical refilling records	3, 6, 9, 12 months
**PrEP discontinuation**	120 days without TDF-FTC in possession based on medical refilling records.	3, 6, 9, 12 months
**PrEP reinitiation**	Number of participants who discontinued TDF-FTC according to the study definition and then successfully restart PrEP, based on self-report and medical refilling records.	3, 6, 9, 12 months
**Secondary outcomes**		
**HIV testing**	Frequency of participants, defined as the number of participants who reported being tested for HIV during the previous three months	3, 6, 9, 12 months
**Sexual and mental health mini-app use**	Frequency of participants, defined as the number of participants who reported using the mini-app in the past three months to receive information about sexual and mental health comparing their pre-intervention and post-intervention engagement	3, 6, 9, 12 months
**PrEP self-efficacy**	Frequency of participants, defined as number of participants who had an increase in PrEP self-efficacy when comparing their pre-intervention and post-intervention self-efficacy	3, 6, 9, 12 months
**HIV incidence**	Frequency of participants, defined as number of participants who reported who have newly diagnosed with HIV in the previous three months	3, 6, 9, 12 months

Secondary outcomes will include HIV testing rate, mHealth use, self-efficacy assessment, and HIV incidence.

### Timeline

The study will span approximately 15 months (Table 1). The first 3 months will be the preparation phase. A crowdsourcing open call will be promoted and held to generate an initial crowdsourced intervention 1.0. Interventions 2.0, 3.0, 4.0 will be developed during the trial with inputs from the ongoing open call. The following 12 months will be the intervention phase. The intervention will be sequentially rolled out in each of the four clusters following the stepped-wedge design. Participants will be surveyed at baseline and every 3 months thereafter. By the 15th month, all participants will have received the intervention for a 3-month interval and the final follow-up survey will be conducted. The SESH research group will manage and oversee intervention progression.

### Data collection activities

In accordance with the demonstration study design, participants in the stepped-wedge design will undergo clinical monitoring for assessment of adherence and side effects every month for the first 3 months after PrEP initiation and every 3 months starting from the 4th month per current US CDC and WHO guidelines, provided by their respective study hospital. All study participants will be required to complete clinical visits at their respective study hospital for PrEP initiation (month 0) as well as for follow-up at months 1, 2, 3, 6, 9 and 12 (Table 1). Baseline survey and follow-up surveys will be administrated through a professional Chinese online survey engine Sojump (Sojump, Shanghai, China) that has been widely used in China-based research studies. Research staff will monitor the survey responses and upload to a secure Dropbox account on a weekly basis.

### Follow-up plan

During study planning in the first half of 2020, reports have emerged regarding the adverse impact of the COVID-19 pandemic on sexual health for people at risk of HIV, including fewer HIV tests, STI screens and less consistent PrEP refills. We have included questions related to the impact of COVID-19 on sexual behaviors and PrEP readiness to our screening survey, and we will monitor accrual and adherence to clinic visits and PrEP with additional caution. As needed, we will incorporate changes in the protocol as required by local pandemic patterns in Hubei and Guangdong provinces.

### Data management

All data from baseline and follow-up surveys are anonymous and are submitted directly into computers and transmitted securely using SSL (TLS) 128-bit encryption. Data will be located password-protected on Sojump and will only be accessed by research group members. Data can be readily downloaded and converted to the format of commercially available statistical software. Survey responses will be kept separately from participants’ contact information; the two files will be linked with a non-descript identifier that is encrypted and password-protected. An independent external advisory committee consisting of STI experts has been formed. The committee will meet periodically to review and evaluate data collection and study progress.

### Analysis

The primary outcome will be evaluated at the end line. We will examine a hypothesis that a crowdsourced intervention can facilitate PrEP adherence. As this is a binary outcome with a small number of clusters, we will use generalized linear mixed models (GLMM) for the primary outcome analysis [31]. It is appropriate to assume that the intervention effect is common across clusters as the intervention will be implemented to all clusters directly by CBO staff. The model will include intervention status and time as fixed effects given the potentially increasing loss-to-follow-up. The estimated intervention effects will be reported with 95%CIs and *p* values.

All secondary outcomes will be binary (continuous variables will be categorized into binary variables). Similar analysis will be conducted for secondary outcomes including proportion of people who test for HIV in the past 3 months, proportion of mhealth use, proportion of people who feel confident in their self-efficacy, and HIV incidence. Descriptive data will be used to summarize clusters.

### Ethical approvals

The study was reviewed and approved by the Institutional Review Boards at the University of Chicago, USA (IRB#20–1388), the Guangzhou Eighth People’s Hospital, China (IRB#202043176) and the Guangdong Provincial Dermatology Hospital, China (IRB#2020031).

## Discussion

Although an increasing number of interventions have been implemented to facilitate PrEP adherence, efforts to promote PrEP adherence in China are scarce. Suboptimal PrEP uptake among key populations, the lack of effective PrEP service delivery models, and insufficient community engagement in PrEP initiatives may have hindered the development of such interventions [32]. The study design addressed these obstacles by using crowdsourcing to generate social media-based intervention materials and embedding the study design in a demonstration project that engages stakeholders in the healthcare system and the community. Crowdsourcing and mobile health interventions can be powerful vehicles to reach, engage, educate and retain sizable key populations on PrEP in a short period of time, with potentials of encouraging behavioral change regarding PrEP uptake and adherence [14, 33]. The inputs from the community and the public contribute to a social media-based intervention that will be further evaluated in a real-world PrEP delivery model. Early engagement and ongoing partnership with key stakeholders in this PrEP delivery model may help identify and address local challenges, leverage the existing infrastructure to solve the problems, and inform evidence-based scale-up with identified resources [14, 34]. We believe these qualities are crucial to improve PrEP uptake and adherence among key populations in China, and that this stepped-wedge design may be able to contribute to local efforts.

Our study has limitations. First, all behavioral measures will be self-reported, which may introduce desirability bias. However, all surveys will be computerized and self-administered, which can reduce the strength of this bias. We will also randomly select 120 participants in Guangzhou to count pills to measure their adherence to PrEP. Triangulating self-reports with this measurement can facilitate validation. Second, the use of social media for open call promotion and intervention implementation may overlook individuals who lack access to online tools. Although social media will be the main venue for study promotion, we will also recruit participants through in-person outreach and chain referral. In addition, intervention may be implemented at the clinical sites. Incorporating those measures may mitigate the impact of the bias.

Our study may help to address important policy and research questions. The study outcomes may help to guide policy and intervention practices among community-based organizations and medical system regarding facilitating adherence to PrEP uptake among key populations. Moreover, community engagement and practical knowledge generated from developing and implementing a crowdsourced intervention may be applicable for future efforts to scale-up programs related to PrEP uptake, retention, and adherence.

## Trial status

At the time of this draft, intervention development and participant recruitment have begun. Study outcomes, data cleaning, and analysis are pending. The study is registered in Clinical Trial databases in China and US (ChiCTR2100048981, NCT04754139).

## Supplementary Information

**Additional file 1.**

## Data Availability

The datasets used and/or analyzed during the current study are available from the corresponding author on reasonable request.

## Abbreviations

PrEP: Pre-exposure prophylaxis
HIV: Human immunodeficiency virus
GBMSM: Gay, bisexual and other men who have sex with men
CI: Confidence interval
MSM: Men who have sex with men
TDF-FTC: Tenofovir/emtricitabine
STI: Sexually transmitted infections
HBV: Hepatitis B virus
RCT: Randomized controlled trial
ICC: Intra-cluster correlation coefficient
CBO: Community-based organization
HCV: Hepatitis C virus
SESH: Social Entrepreneurship to Spur Health
US: the United States
CDC: Centers for Disease Control and Prevention
WHO: World Health Organization
COVID-19: Coronavirus Disease 2019

## References

[1] Dong MJ, Peng B, Liu ZF, Ye QN, Liu H, Lu XL (2019). The prevalence of HIV among MSM in China: a large-scale systematic analysis. BMC Infect Dis.

[2] National Health and Family Planning Commission of the People’s Republic of China (2015). 2015 China AIDS response Progress report.

[3] Wu Z, Chen J, Scott SR, McGoogan JM (2019). History of the HIV epidemic in China. Current HIV/AIDS Rep.

[4] National Center for AIDS/STD Control and Prevention (2019). China CDC annual report of China national HIV/STD/HCV comprehensive prevention and treatment programs in 2018.

[5] Dong Y, Wang L, Burgner DP, Miller JE, Song Y, Ren X (2020). Infectious diseases in children and adolescents in China: analysis of national surveillance data from 2008 to 2017. BMJ.

[6] McCormack S, Dunn DT, Desai M, Dolling DI, Gafos M, Gilson R (2016). Pre-exposure prophylaxis to prevent the acquisition of HIV-1 infection (PROUD): effectiveness results from the pilot phase of a pragmatic open-label randomised trial. Lancet.

[7] Molina JM, Charreau I, Spire B, Cotte L, Chas J, Capitant C (2017). Efficacy, safety, and effect on sexual behaviour of on-demand pre-exposure prophylaxis for HIV in men who have sex with men: an observational cohort study. Lancet HIV.

[8] Laurent C, Dembélé Keita B, Yaya I, Le Guicher G, Sagaon-Teyssier L, Agboyibor MK (2021). HIV pre-exposure prophylaxis for men who have sex with men in west Africa: a multicountry demonstration study. Lancet HIV.

[9] Mugo PM, Sanders EJ, Mutua G, van der Elst E, Anzala O, Barin B (2015). Understanding adherence to daily and intermittent regimens of Oral HIV pre-exposure prophylaxis among men who have sex with men in Kenya. AIDS Behav.

[10] Haberer JE (2016). Current concepts for PrEP adherence in the PrEP revolution: from clinical trials to routine practice. Curr Opin HIV AIDS.

[11] Yan L, Yan Z, Wilson E, Arayasirikul S, Lin J, Yan H (2021). Awareness and willingness to use HIV pre-exposure prophylaxis (PrEP) among trans women in China: a community-based survey. AIDS Behav.

[12] Wang Z, Mo PKH, Ip M, Fang Y, Lau JTF (2020). Uptake and willingness to use PrEP among Chinese gay, bisexual and other men who have sex with men with experience of sexualized drug use in the past year. BMC Infect Dis.

[13] Qu D, Zhong X, Xiao G, Dai J, Liang H, Huang A (2018). Adherence to pre-exposure prophylaxis among men who have sex with men: a prospective cohort study. Int J Infect Dis.

[14] Paul ME, Castillo M, Emmanuel P, Bauermeister JA, Mena LA, Sullivan PS (2021). Scale up mHealth HIV interventions: site and public health perspectives and lessons learned from P3. mHealth.

[15] Songtaweesin WN, Kawichai S, Phanuphak N, Cressey TR, Wongharn P, Saisaengjan C (2020). Youth-friendly services and a mobile phone application to promote adherence to pre-exposure prophylaxis among adolescent men who have sex with men and transgender women at-risk for HIV in Thailand: a randomized control trial. J Int AIDS Soc.

[16] Haberer JE, Bukusi EA, Mugo NR, Pyra M, Kiptinness C, Oware K (2021). Effect of SMS reminders on PrEP adherence in young Kenyan women (MPYA study): a randomised controlled trial. Lancet HIV.

[17] Celum CL, Gill K, Morton JF, Stein G, Myers L, Thomas KK (2020). Incentives conditioned on tenofovir levels to support PrEP adherence among young south African women: a randomized trial. J Int AIDS Soc.

[18] Tucker JD, Day S, Tang W, Bayus B (2019). Crowdsourcing in medical research: concepts and applications. PeerJ..

[19] WHO/TDR and SESH in collaboration with the Social Innovation in Health Initiative. Crowdsourcing in Health and Health Research: A Practical Guide. https://www.who.int/tdr/publications/year/2018/crowdsourcing-practical-guide/en/2018. Accessed Aug 2018.

[20] Wang C, Han L, Stein G, Day S, Bien-Gund C, Mathews A (2020). Crowdsourcing in health and medical research: a systematic review. Infect Dis Poverty.

[21] Tang W, Wei C, Cao B, Wu D, Li KT, Lu H (2018). Crowdsourcing to expand HIV testing among men who have sex with men in China: a closed cohort stepped wedge cluster randomized controlled trial. PLoS Med.

[22] Tucker JD (2017). Crowdsourcing to promote HIV testing among MSM in China: study protocol for a stepped wedge randomized controlled trial. Trials.

[23] Ren C, Tucker JD, Tang W, Tao X, Liao M, Wang G (2020). Digital crowdsourced intervention to promote HIV testing among MSM in China: study protocol for a cluster randomized controlled trial. Trials.

[24] Tang W, Ritchwood TD, Wu D, Ong JJ, Wei C, Iwelunmor J (2019). Crowdsourcing to improve HIV and sexual health outcomes: a scoping review. Curr HIV/AIDS Rep.

[25] Li C, Xiong Y, Muessig KE, Tang W, Huang H, Mu T, et al. A community-engaged mHealth intervention to increase uptake of HIV pre-exposure prophylaxis (PrEP) among gay, bisexual and other men who have sex with men in China: study protocol for a pilot randomized controlled trial. BMJ Open. 2022;12(5):e055899. 10.1136/bmjopen-2021-055899. 10.1136/bmjopen-2021-055899 PMC9092176 35537794

[26] Hargreaves JR, Copas AJ, Beard E, Osrin D, Lewis JJ, Davey C (2015). Five questions to consider before conducting a stepped wedge trial. Trials.

[27] Treweek S, Zwarenstein M (2009). Making trials matter: pragmatic and explanatory trials and the problem of applicability. Trials.

[28] Hussey MA, Hughes JP (2007). Design and analysis of stepped wedge cluster randomized trials. Contemp Clin Trials.

[29] Li F, Hughes JP, Hemming K, Taljaard M, Melnick ER, Heagerty PJ (2021). Mixed-effects models for the design and analysis of stepped wedge cluster randomized trials: an overview. Stat Methods Med Res.

[30] Hargreaves JR, Prost A, Fielding KL, Copas AJ (2015). How important is randomisation in a stepped wedge trial?. Trials.

[31] Barker D, D'Este C, Campbell MJ, Mcelduff P (2017). Minimum number of clusters and comparison of analysis methods for cross sectional stepped wedge cluster randomised trials with binary outcomes: a simulation study. Trials.

[32] Xu J, Tang W, Zhang F, Shang H (2020). PrEP in China: choices are ahead. Lancet HIV.

[33] Créquit P, Mansouri G, Benchoufi M, Vivot A, Ravaud P (2018). Mapping of Crowdsourcing in Health: Systematic Review. J Med Internet Res.

[34] Weinfurt KP, Hernandez AF, Coronado GD, DeBar LL, Dember LM, Green BB (2017). Pragmatic clinical trials embedded in healthcare systems: generalizable lessons from the NIH Collaboratory. BMC Med Res Methodol.

