# Supplementary material for: Co-creation using crowdsourcing to promote PrEP adherence in China: study protocol for a stepped-wedge randomized controlled trial
Source: BMC Public Health. 2022 Sep 7;22:1697. doi: 10.1186/s12889-022-14117-5 (PMC9449927; doi:10.1186/s12889-022-14117-5)

Supplement

Summary of the recognized contributions followed by the contributions

| Contribution | Description of content | Core element |
| --- | --- | --- |
| 1 | Emphasizing the effectiveness of PrEP to raise PrEP awareness | Poster |
| 2 | Emphasizing the protective aspects of PrEP to raise awareness | Poster |
| 3 | PrEP-related knowledge: PrEP can be effective to prevent HIV but is unable to prevent STIs | Comics |
| 4 | PrEP-related knowledge: 2+1+1 medication | Poster |
| 5 | PrEP-related knowledge: daily and on-demand medications (including who should take PrEP and how to take PrEP) | Poster |
| 6 | PrEP awareness knowledge: effectiveness, daily and on-demand medications (including who should take PrEP and how to take PrEP) | Poster |

Contribution 1


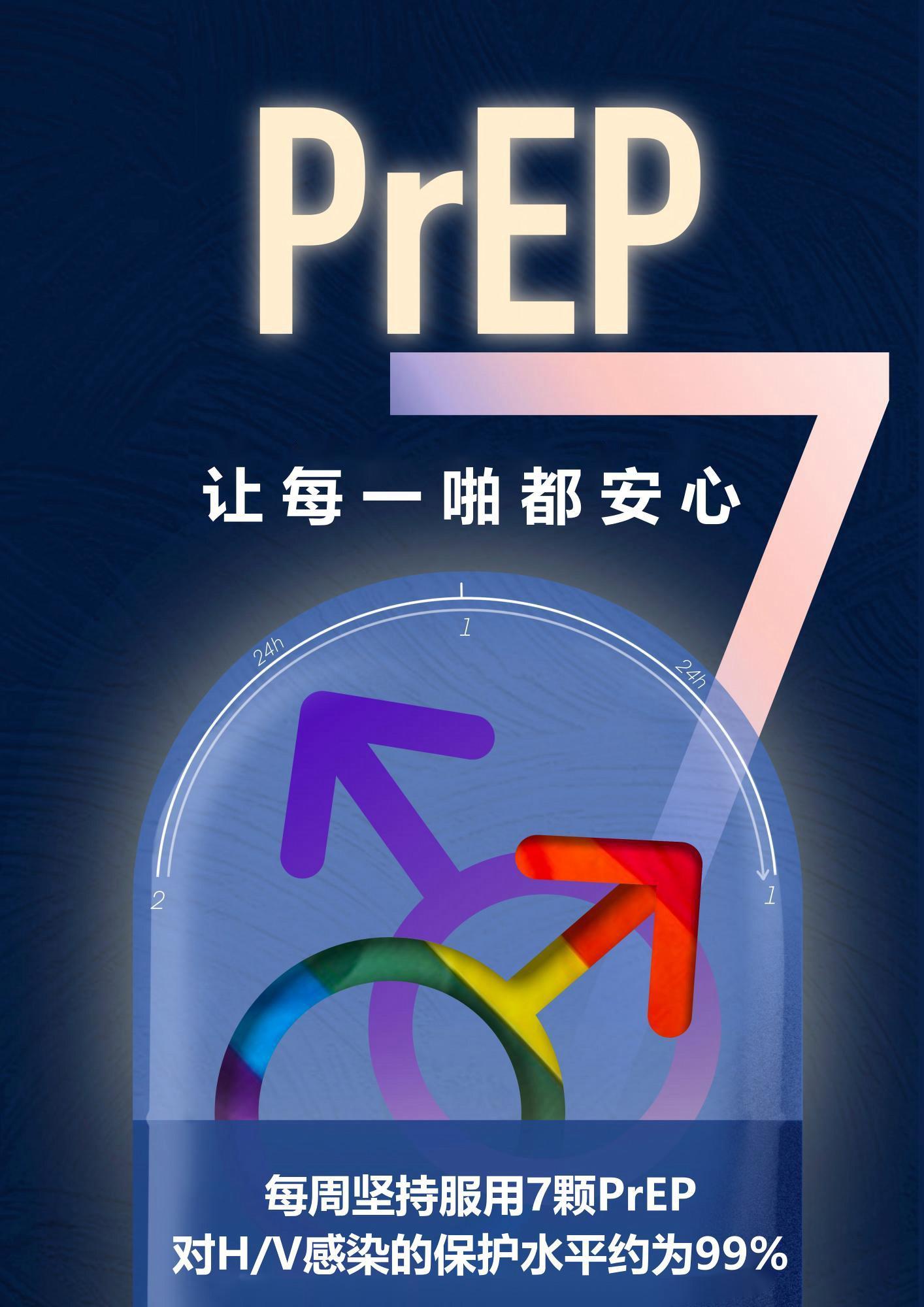


Contribution 2


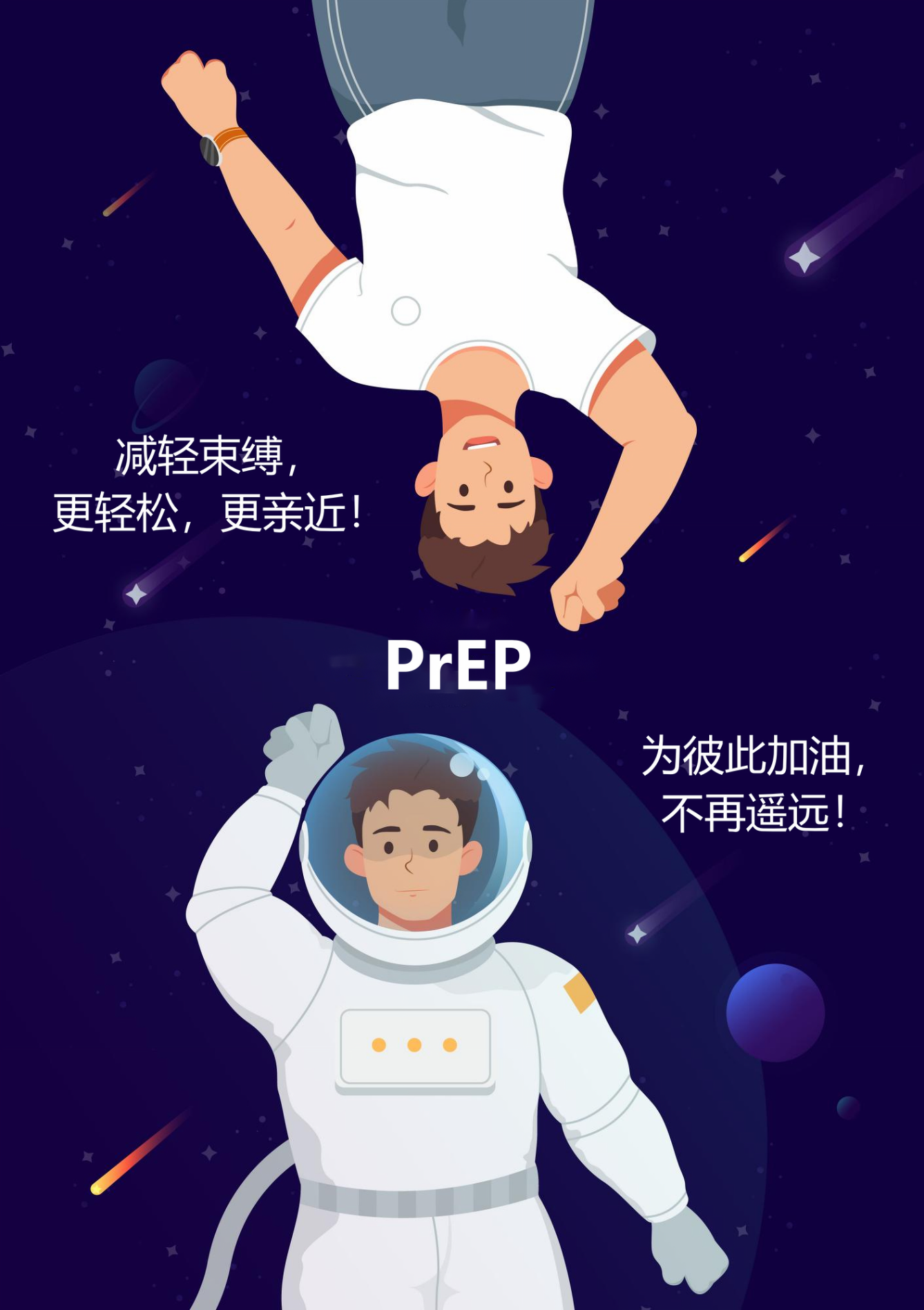


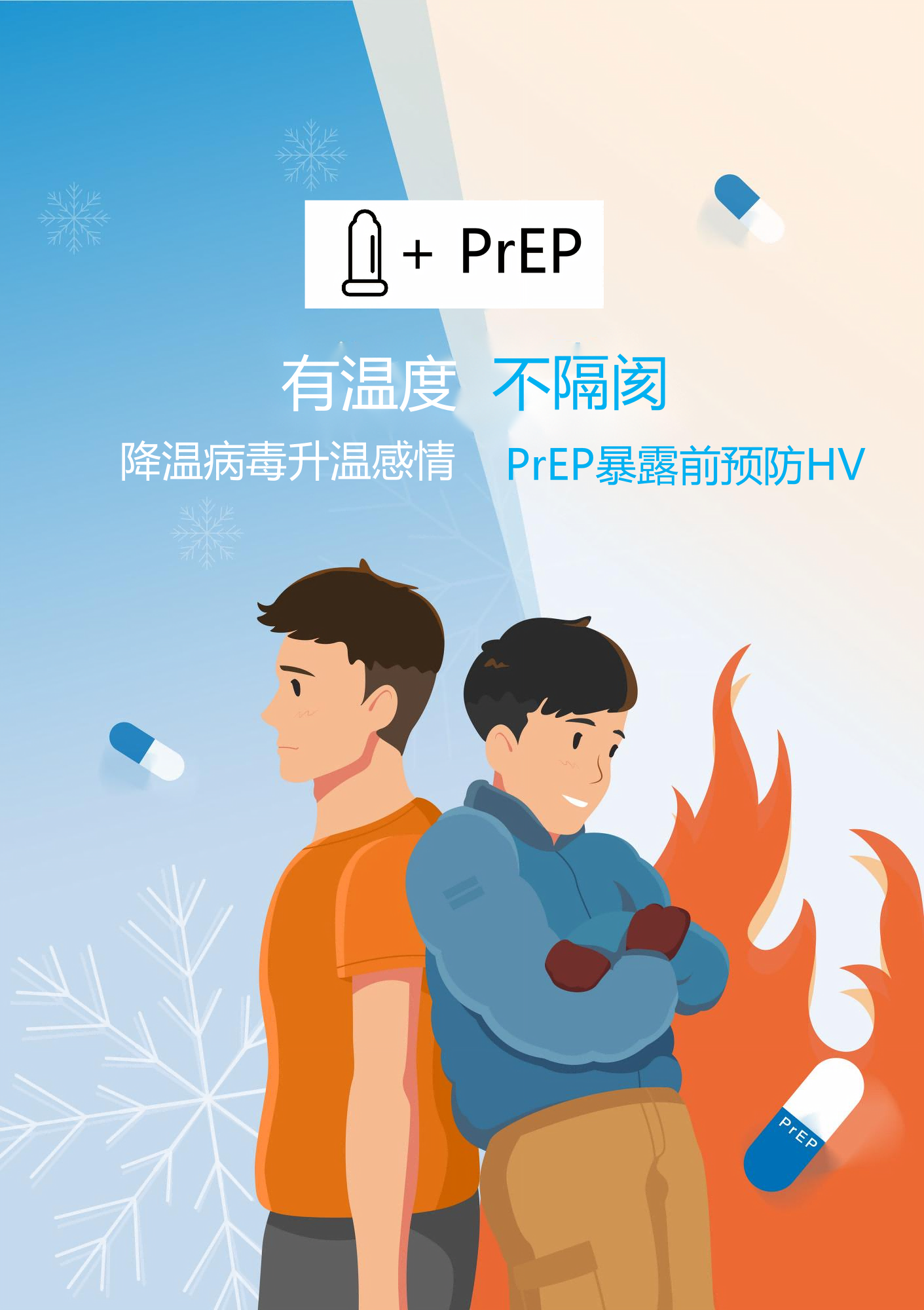

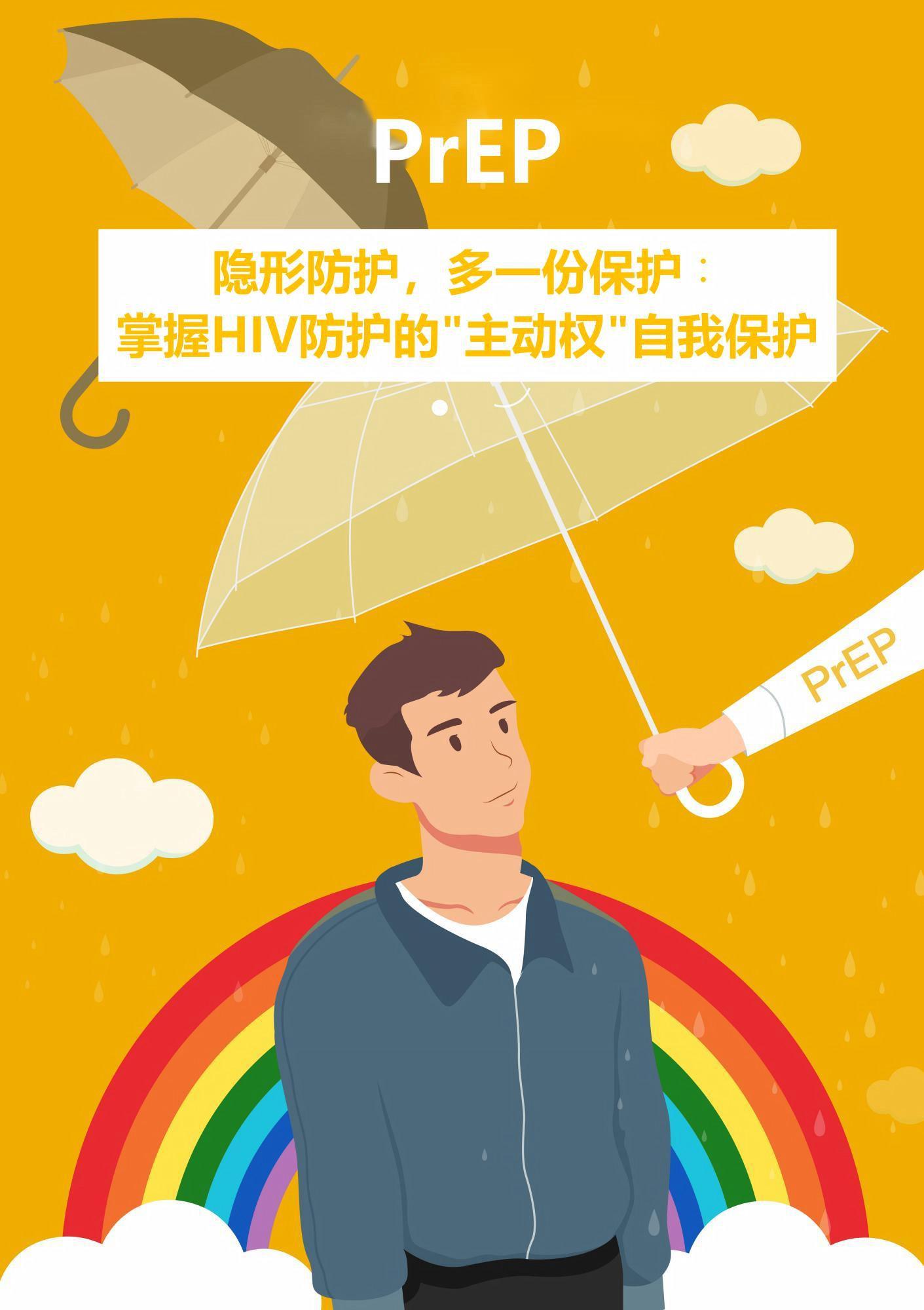


Contribution 3


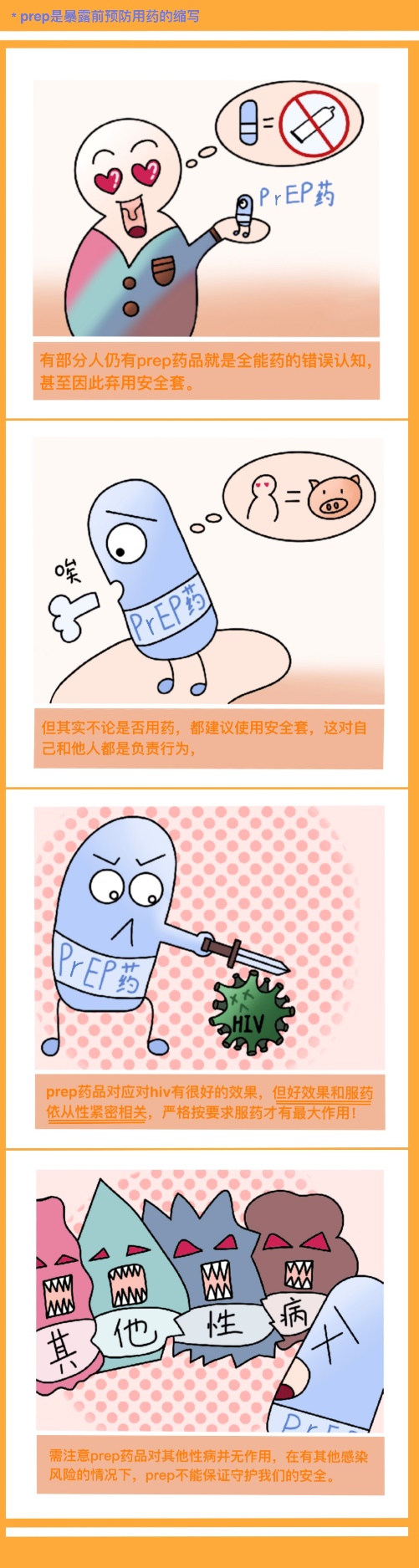


Contribution 4


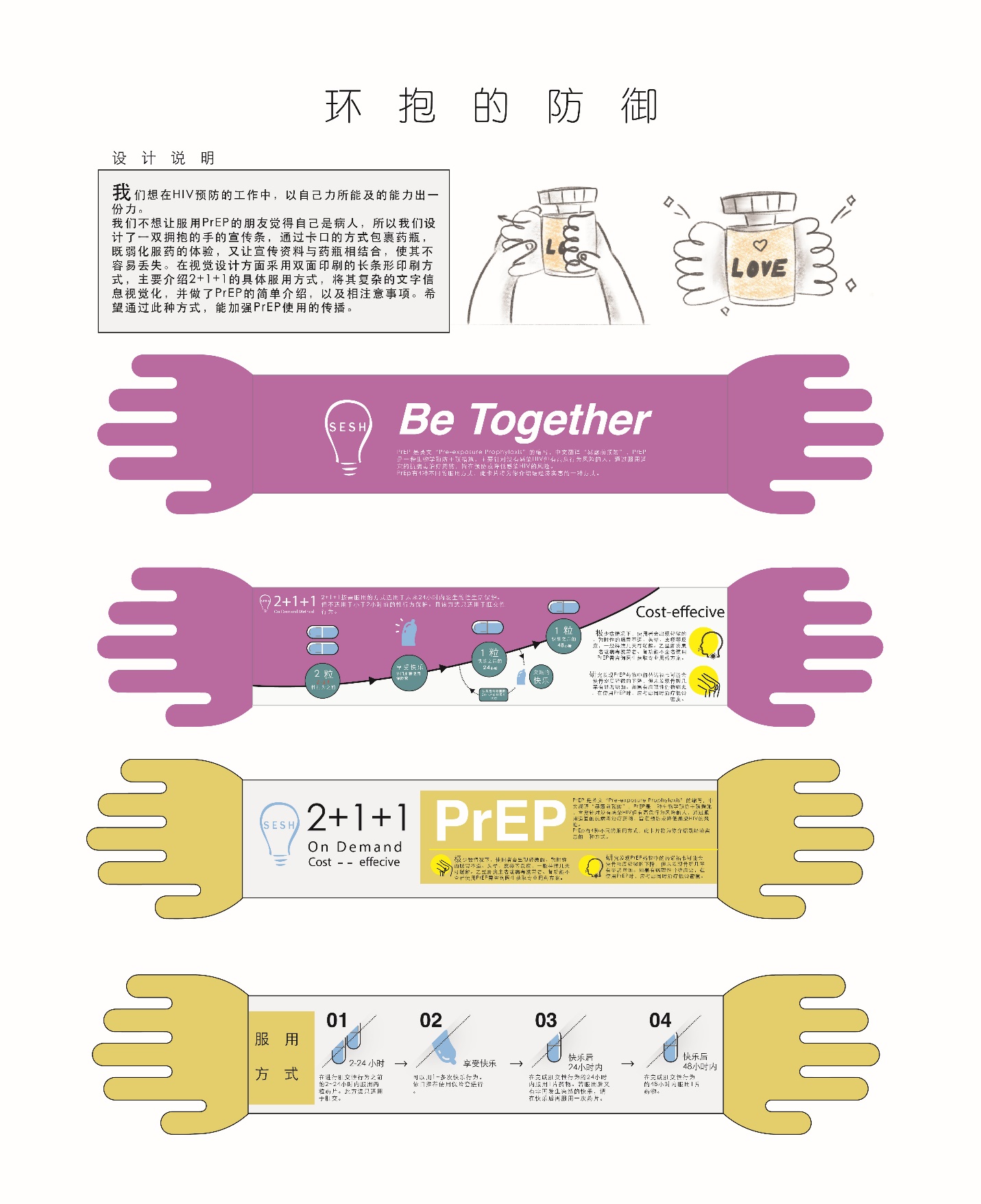


Contribution 5


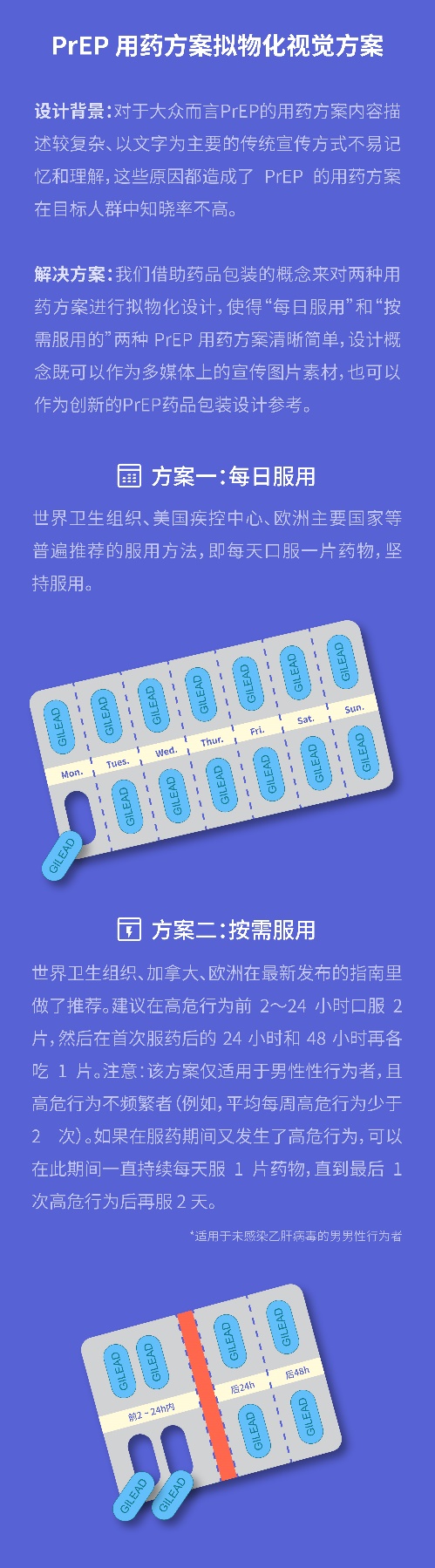


Contribution 6


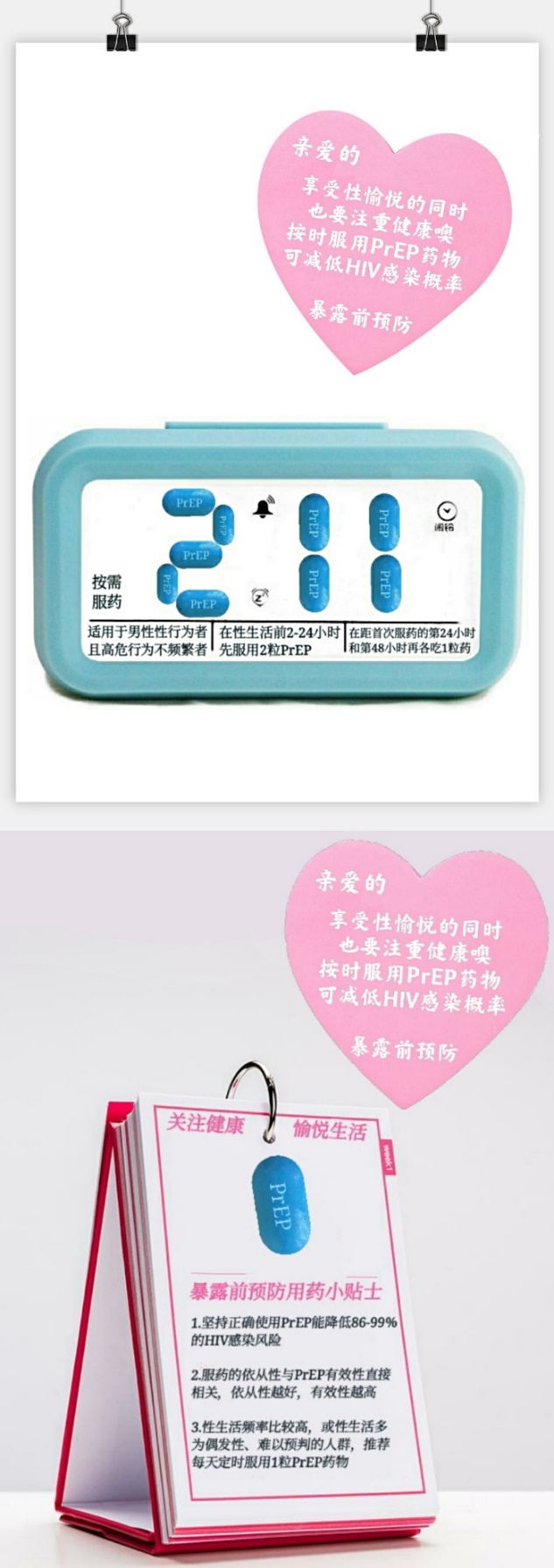

Supplement: Supplementary file 1 — Additional file 1. [file 12889_2022_14117_MOESM1_ESM.docx]
